# 

**Published:** 1894-09-15

**Authors:** 


**Notices of Births, Deaths, and Marriages** are inserted in this column at a charge of 2s. 6d. for an announcement not exceeding four lines.

**Notice to Advertisers and Subscribers.**

All Advertisements, Orders for Copies of The Hospital, and Business Communications should be addressed to **The Publisher, NOT TO THE EDITOR,** The Hospital, 428, Strand, W.C.

The Cost of Subscription, post free, is as follows:—Per week, 2½d.; per quarter, 2s. 8d.; per half-year, 5s. 3d.; per annum, 10s. 6d. Foreign:—Per Annum, 12s. 8d.; payable, in all cases, in advance.

### NOTICE TO CORRESPONDENTS.

All MS., Letters, Books for Review, and other matters intended for the Editor should be addressed The Editor, The Lodge, Porchester Square, London, W.

The Editor cannot undertake to return rejected MS., even when accompanied by stamped directed envelope.

**"Burdett's Hospital Annual," 1894.**

Fifth year of publication. 900 pp. Boards, gilt lettering. A most complete guide to British, Colonial, Indian, and American Hospitals, Dispensaries, Nursing and Convalescent Institutions, and Asylums. Price 5s. Published by The Scientific Press (Limited), 428, Strand, W.C.

**NOTICE.—The Index for Vol. XV. (ending March 31st, 1894) is on sale at the Office of this Journal, price 2½d., post free.**

### SCRAPS AND GLEANINGS.

SEPTEMBER 29th is the day fixed for the Hospital Saturday collection at Worcester.

OWING to the excellent management of the Hospital Saturday Committee and the collectors, the collections at Bournemouth this year amounted to over £107.

IT is proposed to build an isolation hospital for Penzance in the parish of Madron, for which purpose it would be necessary for the town council to borrow £650 from the Local Government Board.

THE Worcester General Infirmary Committee have decided to hold a special meeting to sanction the selling out of Consols to the amount of £1,500 to meet special and current expenses.

THE Northampton Infirmary Sports Committee announced at their annual meeting this year that the sports had resulted in a profit of £162. The committee have now handed over sums exceeding £1,000 to the Infirmary.

THE Cottage Hospital scheme for Tilbury and district has at last assumed a practical form. A permanent committee has been formed, and promises of subscriptions received. The hospital is expected to be in actual operation before long.

THE third annual Church parade of friendly and other societies of Bishopstoke and the immediate neighbourhood has just taken place. The money collected at the meeting was devoted to the funds of the Winchester and Southampton hospitals.

THE committee of St. Mark's Ophthalmic Hospital, Dublin, are urgently soliciting the public for funds to carry on the work of the institution. At the last meeting of the board of governors the banking account was shown to be considerably overdrawn.

A LOCAL doctor has recently called public attention to the urgent need of a home for incurables at Liverpool, preferably in connection with the Royal Infirmary, which cannot burden itself with hopeless cases. It is hoped the appeal will be generously responded to.

THE Medical School of Queen's College, Belfast, is to be substantially improved. The Government have granted a sum of £2,500 for the erection and equipment of a physiological and pathological laboratory. Plans are being prepared and the building will be at once proceeded with.

ON account of the adverse balance in the accounts of the Essex and Colchester Hospital a local practitioner suggests that funds should be raised by means of collection-boxes placed in halls, and collection-cards for the consulting-rooms of local doctors. The deficit this year will amount to £1,000.

AN inquiry is being made in regard to the application of the Commissioners of the Public Baths for the sanction of a loan to reconstruct and alter the present baths at Marylebone. A number of rates payers oppose the scheme on the grounds of excessive outlay without much extra accommodation.

THE kitchen accommodation of the Grimsby and District Hospital has proved itself quite inadequate to the requirements of the institution. Such alterations in this direction as are necessary will be effected in the spring, the funds of the hospital at present not allowing sufficient expenditure for the scheme.

A MEETING was held last week at Mile End under the auspices of the Mile End Old Town Branch of the London Society for the Abolition of Compulsory Vaccination. A resolution was carried pledging the audience not to vote for any candidate for local or political office who upheld the Act for compulsory vaccination.

No definite conclusion is yet arrived at in connection with the proposed joint infectious hospital for Liversedge and Mirfield. The estimated cost of such a building is £7,000. The Government Board have refused to allow smallpox and fever wards to be provided on the same site, and the difficulty is still under discussion.

IT has been proposed to devote certain moneys deposited in the Glamorganshire Bank for a charitable purpose by the late Mr. J. D. Llewelyn and Mr. Grenfell to the purpose of endowing and maintaining gratuitously an in-patient bed in the Swansea Hospital, the same to be called the "Llewelyn and Grenfell" bed.

AT the recent annual meeting of the subscribers to the Hertfordshire Convalescent Home it was shown that the funds were in a most satisfactory condition, and that there is hardly a district in Hertfordshire which has not already sought the assistance of the institution. During the past year 671 patients have passed through the home.

THE annual general meeting of the governors of the Stockton and Thornaby Surgical Hospital was held recently. The report stated that the number of patients treated during the year has been lower than in the three preceding years. A pleasing item in the report of this institution is the increase in the workmen's subscriptions.

GREAT efforts were made this year at Ilfracombe to make Hospital Saturday a success. The Victoria Promenade was crowded at the evening concert, and concerts were also held at several private hotels and boarding-houses, besides many other entertainments in connection with the charity. The total amount collected was over £208.

A BAZAAR is to be held in December, 1895, in the Waverley Market at Edinburgh in aid of the Royal Infirmary, with a view to the extension of the institution. A contribution of four shillings was lately received accompanied by these words: "The writer encloses his first week's wages, with grateful acknowledgment of the kindness received in the surgical ward."

THE Blackburn and East Lancashire Infirmary will have to wait for the projected and much-wanted new laundry and operating theatre, as the present condition of the funds will not admit of more outlay. The secretary's attention has been drawn to the fact of many patients attending the infirmary who can afford to pay for medical advice, and steps are being taken to prevent this.

A NEW opening for philanthropic efforts lies in the suggestion to organise town trips for pauper country children who need an outing as much as London children. A great many of the latter have been able this year to share in the benefits of the Children's Country Holiday Fund, and it is hoped next year to give the same pleasure of a change to children residing in "slums-by-the-sea" by giving them a visit to London.

### THE STUDENT OF MEDICINE.

### APPOINTMENTS.

A. H. Buck, M.R.C.S.Eng., L.R.C.P.Lond., appointed Senior House Surgeon to Sussex County Hospital, Brighton. A. E. Chilcott, M.R.C.S.Eng., L.R.C.P.Lond., appointed Medical Superintendent of the Infirmary of the Parish of St. Pancras. Mr. A. Crawford, appointed Assistant Medical Officer of the Infirmary of the Parish of St. George's-in-the-East. A. T. Collum, M.B.Lond., F.R.C.S.Eng., appointed Assistant-Surgeon to Charing Cross Hospital. A. T. Davies, M.D.Cantab., F.R.C.P. Lond., appointed Physician to the Metropolitan Hospital. W. Duncan, M.D., M.R.C.P., F.R.C.S., appointed Physician to In-Patients at the Chelsea Hospital for Women. Mr. J. Fuller, appointed Medical Officer of the Workhouse of the Bedminster Union. J. J. Joseph, L.R.C.P., L.R.C.S.I., appointed Medical Officer for the No. 2 Battersea District of the Wandsworth and Clapham Union. H. R. Kenwood, M.B.Edin., D.P.H., appointed Medical Officer of Health to the Stoke Newington Sanitary District. A. F. W. King, L.R.C.P., M.R.C.S., appointed Clinical Assistant in the Special Department for Diseases of the Throat at St. Thomas's Hospital (extension). J. W. Laver, L.R.C.P., M.R.C.S., appointed Clinical Assistant in the Special Department for Diseases of the Skin at St. Thomas's Hospital. F. T. Long, M.R.C.S.Eng., L.R.C.P.Lond., appointed Medical Officer and Public Vaccinator for the Colton District of the Ulverston Union. J. H. Marsh, M.R.C.S., L.R.C.P.Lond., appointed Senior House Surgeon to the Macclesfield General Infirmary. C. H. Melland, M.R.C.S., L.R.C.P., appointed Clinical Assistant at the Barnes Convalescent Home at Cheadle of the Manchester Royal Infirmary. W. J. C. Merry, M.B., B.Ch.Oxon., appointed House Surgeon to St. Thomas's Hospital. L. J. Miskin, L.R.C.P., M.R.C.S., appointed House Surgeon to St. Thomas's Hospital. T. G. Nicholson, B.Sc.Lond., L.R.C.P., M.R.C.S., appointed Non-resident House Physician to St. Thomas's Hospital. R. E. Nix, B.A., M.B., B.C.Cantab., L.R.C.P., M.R.C.S., appointed Resident House Physician to St. Thomas's Hospital (extension). J. E. Platt, F.R.C.S.Eng., B.S., M.D.Lond., appointed Resident Surgical Officer to the Manchester Royal Infirmary. S. W. Plummer, M.D.Durh., appointed Medical Officer to the Durham Union Workhouse. F. Radcliffe, M.B., B.Ch.Vict., appointed House Physician to the Manchester Royal Infirmary. J. H. Ray, M.B., B.Ch.Vict., appointed House Surgeon to the Manchester Royal Infirmary. S. W. F. Richardson, B.Sc.Lond., L.R.C.P., M.R.C.S., appointed Clinical Assistant in the Special Department for Diseases of the Ear at St. Thomas's Hospital. A. E. Russell, L.R.C.P., M.R.C.S., appointed Clinical Assistant in the Special Department for Diseases of the Skin at St. Thomas's Hospital. W. Russell, M.B., C.M.Aberd., appointed Resident Medical Officer Netherfield Road Fever Hospital, Liverpool. G. C. Sandford, M.B., C.M.Edin., appointed Assistant Medical Officer to the City Hospital, Birmingham. Mr. J. Stevenson, appointed Assistant Medical Officer at the Infirmary of the Parish of St. Leonard, Shoreditch. R. Fox Symons, L.R.C.P., M.R.C.S., appointed Assistant House Surgeon to St. Thomas's Hospital. P. Thomson, M.B., B.Ch.Vict., appointed House Physician to the Manchester Royal Infirmary. J. K. Warry, M.B.Lond., appointed Medical Officer of Health to the Hackney Sanitary District. F. J. Webb, M.B., B.Ch.Vict., appointed Medical Officer and Public Vaccinator for the Gorton (Sixth) District of the Chorlton Union, Manchester. P. Withers, M.R.C.S., L.R.C.P., appointed House Surgeon to the Manchester Royal Infirmary. Mr. W. M. Woodhouse, appointed Junior Assistant Medical Officer of the Infirmary of the Parish of Kensington. R. Young, M.D.Durh., M.R.C.S.Eng., appointed Medical Officer of the Workhouse and Schools of the Oldham Union. Mr. W. Ledingham Christie, M.D., F.R.C.S., has been appointed House Surgeon at the Bristol Hospital for Sick Children and Women.

### VACANCIES.

The following vacancies are announced this week, the amount of salary in each case being given after the name of the institution:

### Resident Medical Officers.

Bethlem Hospital.—Two Resident Clinical Assistants. Apartments, rations, and attendance provided. Applications, endorsed "Clinical Assistantship," to the Treasurer by October 1st.

Cambridge University.—John Lucas Walker Studentship. Applications to Professor Roy, New Museums, Cambridge, by September 30th.

Royal Surrey County Hospital, Guildford.—Assistant House Surgeon. No salary, but board, residence, and laundry found. Applications and testimonials to the Honorary Secretary, by September 17th.

THE STUDENT OF MEDICINE—*continued.*

Cornwall County Asylum, Bodmin.—Junior Assistant Medical Officer; unmarried. Salary to commence at £100 per annum, increasing annually to £120 per annum, with board, lodging, washing, and attendance. Applications to the Medical Superintendent by September 15th.

Great Yarmouth Hospital.—House Surgeon; must be doubly qualified. Salary £90 per annum, with board and lodging. Applications and testimonials to Richard F. Ferrier, Honorary Secretary, by September 22nd.

Royal United Hospital, Bath.—House Surgeon. Candidates must be M.R.C.S. Eng. and registered. Salary £60 per annum, with board, lodging, and washing. Appointment for one year. Applications and testimonials to W. Stockwell, Secretary-Superintendent, by September 18th.

Sussex County Hospital, Brighton.—House Physician; unmarried and under 30 years of age. Salary £60 per annum, with board, residence, and washing, rising to £80. Applications and testimonials to Lieutenant-General E. F. Bourchier, Secretary, by September 19th.

West London Hospital, Hammersmith Road, W.—House Physician and House Surgeon. Appointments for six months. Board and lodging provided. Applications to R. J. Gilbert, Secretary Superintendent, by September 19th.

**Non-Resident Medical Officers.**

Coventry Provident Dispensary.—Surgeon; must be fully qualified medical practitioner. Applications and testimonials to W. Sleath, Honorary Secretary, by September 17th.

Dental Hospital of London, Leicester Square. — Assistant Dental Surgeon. Must be Licentiate of Dental Surgery of one of the licensing bodies recognised by the General Medical Council. Applications and testimonials to J. Francis Pink, Secretary, by October 8th.

Plymouth Public Dispensary.—Physician's Assistant; doubly qualified. Salary £60 per annum. Appointment for one year, but eligible for re-election. Applications to the Honorary Secretary before September 18th.

The General Infirmary at Gloucester and Gloucestershire Eye Institution.—Assistant Surgeon; must be F.R.C.S. or M.R.C.S. Eng., or F.R.C.S. or L.R.C.S. of Ireland or Edinburgh, or graduate of University recognised by the General Medical Council. Applications and testimonials to Henry T. Pike, Secretary, by October 10th.

New Hospital for Women, 144, Euston Road, N.W.—Female Assistant Physician; appointment for two years. Also two Clinical Assistants for Out-patient Department; appointment for one year. Applications and testimonials to Margaret M. Bagster, Secretary, by September 26th.

### Medical Appointments.

SUSSEX COUNTY HOSPITAL.

London: The Scientific Press (Limited), 428, Strand, W.C.

Printed by W. H. and L. Collingridge, City Press, 148 and 149, Aldersgate Street, London, E.C.; and Published by the Proprietors, The Scientific Press (Limited), at 428, Strand, London, W.C.

To be had of all Booksellers and Newsagents in the United Kingdom, and at the Railway Bookstalls of Messrs. W. H. Smith and Son.

### RECENT EDITIONS OF STANDARD BOOKS.

*Sixth Edition, profusely illustrated with over* 100 *cuts ; carefully revised, cloth gilt, crown* 8*vo., about* 400 *pp., price* 3*s.* 6*d.* (*post free*).

### The Theory and Practice of Nursing.

**A Text = Book for Nurses.**

By PERCY G. LEWIS, M.D., M.R.C.S., L.S.A.

Always a standard work on Nursing; it now occupies the position of a classic, being used throughout the world in Training Schools and by Private Students.

"The fact that the first edition of this nursing manual has been quickly exhausted in very little more than a year shows that it well supplies an ever-increasing demand for knowledge amongst nurses. . . . We have read the book with interest, and can warmly recommend it as a Nursing Manual. . . . The section on the Administration of Medicine, on Fractures and Splint Making on the Female Pelvic Organs, and on Convalescence, are especially good. The illustrations are well executed and plentiful. The Hospital Nurse, for whom the book is especially written, will find it full of useful hints and information."—*Birmingham Medical Review.*

". . . Nurses will find the book full of interest and instruction, which cannot fail to assist them in their work. . . . Dr. Lewis is successful in pointing out the main duties a nurse has to perform at the bedside, and in warning her what not to do, which is often more important."—*British Medical Journal* (second notice).

"Dr. Lewis, through many years of hospital experience, has earned the right to speak with authority upon the subject of nursing. The chapters on surgery call for special praise. No professional nurse can afford to dispense with the work."—*The Queen.*

"The name of the author of this valuable little book, which ought to be in every house, is a sufficient recommendation. It is written in a most lucid manner, and any one who consults it, and follows out the directions given in it, cannot fail to bring help and comfort to a sick bed."—*Lady's Pictorial.*

*Second and Revised Edition, demy* 16*mo.* (*suitable for the apron pocket*), *bound in terra cotta cloth boards,* 140 *pp., price* 2*s.; handsomely bound in leather, gilt, price* 2*s.* 6*d. nett.*

### The Nurse's Dictionary of Medical Terms and Nursing Treatment.

**Compiled for the use of Nurses.**

By HONNOR MORTEN.

Containing descriptions of the Principal Medical and Nursing Terms and Abbreviations, Instruments, Drugs, Diseases, Accidents, Treatments, Physiological Names, Operations, Foods, Appliances, &c., &c., encountered in the Ward or Sick-room.

"This is a very useful little book for reference purposes, and some such should be at the disposal of every nurse, more especially in the early days of training. It comprises not only what its name implies, but a description of the common abbreviations used, of many instruments, drugs, &c."—*The Birmingham Medical Review.*

"Honnor Morten has compiled a book for the use of nurses which is likely to be of service. 'The Nurse's Dictionary' explains medical terms and abbreviations, besides giving much information regarding everything to be encountered in the ward or sick-room. Brevity and simplicity are the keynotes of this little volume."—*The Morning Post.*

"This is a small and convenient manual. . . . A portable handbook of this kind which can be consulted at a moment's notice will prove a boon. . . . The plan of it is to be commended. Where mere defini ions alone are not thought enough, particulars are given in a very compendious form."—*Queen.*

"We are glad to see a second enlarged and much improved edition of Miss Morten's useful and convenient 'Nurse's Dictionary' (The Scientific Press), which will be found a necessary companion, not only for the sick-room but the nursery."—*St. James's Gazette.*

*Second Edition, demy* 8*vo., profusely illustrated, nearly* 200 *pp., cloth. Important Text-book for Asylum Attendants, price* 2*s.* 6*d.* (*post free*).

### Mental Nursing.

A Text-book specially designed for the instruction of Attendants on the Insane. By William Harding, M.B. The want of a complete book for the instruction of Asylum Attendants has been long felt, and it is with confidence that the publishers recommend this work to the managers of all Institutions for the Insane.

*Demy* 8*vo.,* 200 *pp., cloth boards, copiously illustrated, price* 3*s.* 6*d.* (*post free*).

### Surgical Ward=work and Nursing.

By ALEXANDER MILES, M.D. (Edin.), C.M., F.R.C.S.E.

A practical manual of clinical instruction for Nurses and Students in the Wards. Concisely, though comprehensively, treated.

IMPORTANT NEW HAND-BOOK OF OPHTHALMIC NURSING.

200 *pp., crown* 8*vo., profusely illustrated with original drawings. List of Instruments required, and a Glossary of Terms, now ready, price* 3*s.* 6*d.*

### Ophthalmic Nursing.

By SYDNEY STEPHENSON, M.B., F.R.C.S.E., Surgeon to the Ophthalmic School, Hanwell, W., &c., &c. This is a valuable contribution to Nursing literature on a subject hitherto never handled with special reference to Nursing. On account of its compact and clear arrangement, and its numerous illustrations, it is especially recommended to the use of the Nursing Profession.

*Third Edition, enlarged and partly re-written, cloth gilt, crown* 8*vo., about* 200 *pp., price* 2*s.* 6*d.*

### Hospital Sisters and their Duties.

By EVA C. E. LUCKES, Matron to the London Hospital.

The favourable reception accorded to the first two editions of this useful and interesting work, and the regular demand for copies of it, encourage the publishers to place this third edition, greatly improved and enlarged, before the Institutions. The experience and position of its author entitle it to authority, and every Matron, Sister, or Nurse who aspires to become a Sister should read the advice and information contained in its pages.

*Second Edition, demy* 8*vo.,* 200 *pp., profusely illustrated with Copyright Portraits and Drawings, price* 2*s.* 6*d.* (*post free*).

### How to Become a Nurse: And How to Succeed.

A complete guide to the Nursing Profession for those who wish to become Nurses, and a useful book of Reference for Nurses who have completed their training and seek employment. Compiled by Honnor Morten (Author of "Sketches of Hospital Life," "The Nurse's Dictionary," &c.

London: THE SCIENTIFIC PRESS (Limited), 428, Strand, W.C.

